# Balancing Bleeding and Thrombosis in Dental Management of Patients on Novel Oral Anticoagulants: A Narrative Evidence Synthesis

**DOI:** 10.7759/cureus.98373

**Published:** 2025-12-03

**Authors:** Rama Shankar Choudhary, Nishit Kumar, Mridu Dubey, Ballamudi Sarat Ravi Kiran

**Affiliations:** 1 Department of Dentistry, Tata Main Hospital, Jamshedpur, IND; 2 Department of Oral and Maxillofacial Surgery, Government Dental College and Hospital, Jamnagar, IND

**Keywords:** dentistry, direct acting oral anticoagulant, hemorrhage, review, thrombosis

## Abstract

This narrative review explored the perioperative hemostatic challenges posed by novel oral anticoagulants (NOACs) in dental practice, synthesizing evidence on strategies to equilibrate bleeding and thrombotic risks during oral surgical interventions. A structured literature search across major databases, supplemented by manual searching of guidelines and conference proceedings from inception up to October 2025, prioritized high-quality trials, cohorts, and consensus documents while excluding isolated case reports. Pharmacokinetic variability among agents, such as rapid renal clearance of dabigatran versus mixed hepatic-biliary pathways for factor Xa inhibitors, guides precise timing of dose omission, with renal function emerging as a critical modifier. Risk stratification integrates thrombotic propensity via clinical scoring with procedural bleeding potential and classifies interventions from low (supragingival scaling) to high (extensive flap surgery). Evidence robustly supports uninterrupted anticoagulation for minor procedures when paired with local hemostatics; topical tranexamic acid, absorbable matrices, and primary closure effectively contain postoperative oozing. Moderate- to high-risk surgeries benefit from trough-timed interruptions, typically one skipped dose for twice-daily agents and two for once-daily, combined with minimally invasive techniques including flapless implantology, piezoelectric osteotomy, and laser coagulation. Reversal agents, including idarucizumab for dabigatran and andexanet alfa for apixaban, edoxaban, and rivaroxaban, remain emergency-only tools because of their logistical and prothrombotic constraints. Multidisciplinary preoperative consultation refines decisions in complex cases. Despite consistent observational safety signals, randomized dental-specific data are limited, bleeding definitions are heterogeneous, and the long-term thrombotic consequences of brief pauses are underexplored. Real-world confounders, such as renal decline, polypharmacy, and inflammation, are underrepresented. Nonetheless, this narrative review enables safe continuation in most scenarios, minimizing morbidity through pharmacokinetic awareness, local hemostatic rigor, and surgical conservatism. Future prospective registries with standardized outcomes are essential to validate interruption thresholds and adjunctive modalities in this expanding anticoagulation population.

## Introduction and background

The introduction of novel oral anticoagulants (NOACs), also referred to as direct oral anticoagulants (DOACs), has transformed the management of thromboembolic disorders, including nonvalvular atrial fibrillation, venous thromboembolism, and secondary prevention following orthopedic surgery [1]. These agents, including the direct thrombin inhibitor dabigatran and the factor Xa inhibitors rivaroxaban, apixaban, and edoxaban, provide several advantages over traditional vitamin K antagonists, such as warfarin. Their predictable pharmacokinetic and pharmacodynamic profiles eliminate the need for routine coagulation monitoring, reduce drug-food interactions, and simplify dosing regimens [1,2]. Large-scale randomized controlled trials have established their noninferiority or superiority in preventing thromboembolic events, while demonstrating comparable or lower rates of intracranial hemorrhage [3,4]. Consequently, NOACs have become the preferred anticoagulant class in many clinical scenarios, leading to their widespread adoption among patients requiring long-term thromboprophylaxis [1-4].

In the field of dentistry, the increasing prevalence of NOAC therapy presents a complex clinical challenge [5]. Dental procedures ranging from simple extractions to complex implant placements and periodontal surgeries inherently disrupt vascular integrity, creating a tension between the risk of perioperative bleeding and the potential for thrombotic complications if anticoagulation is interrupted [6]. Unlike warfarin, NOACs lack standardized reversal protocols in many settings, and their rapid onset and offset complicate perioperative decision making [7]. Dentists have to carefully manage bleeding without clear, universally accepted lack of procedure-specific dental guidelines and often rely on institutional protocols or expert opinions. The decision to continue, temporarily withhold, or bridge therapy involves individualized risk assessment, factoring in patient-specific thromboembolic propensity, procedural bleeding potential, and renal function, which significantly influences NOAC clearance.

This narrative review critically appraises the evolving research on evidence-based interventions to maintain hemostatic balance in NOAC patients undergoing dental treatment. By synthesizing data from clinical trials, observational studies, and consensus statements, we examined strategies such as the optimal timing of drug interruption, application of local hemostatic measures, adoption of minimally invasive surgical techniques, and judicious use of specific reversal agents when indicated. The goal is to provide clinicians with a practical, evidence-based framework to minimize both hemorrhagic and thrombotic morbidity in this growing patient population.

## Review

Search methodology

This narrative review was conducted through a structured yet flexible literature search to capture key evidence on the perioperative management of patients taking NOACs in dentistry. Electronic databases, including PubMed/MEDLINE, Embase, Cochrane Library, and Scopus, were searched from inception to October 2025 using combinations of MeSH terms and keywords: (“direct oral anticoagulants” OR “NOAC” OR “DOAC” OR dabigatran OR rivaroxaban OR apixaban OR edoxaban) AND (dentistry OR “oral surgery” OR extraction OR implant OR periodontal) AND (bleeding OR hemorrhage OR thrombosis OR perioperative OR interruption OR reversal OR hemostasis). No language restrictions were applied and non-English articles with English abstracts were screened for relevance.

Additional sources included hand-searching reference lists of seminal reviews, clinical guidelines from the American Dental Association, European Society of Cardiology, and International Society on Thrombosis and Hemostasis, and conference abstracts from major dental and hematology meetings (such as American Association of Oral and Maxillofacial Surgeons, International Association for Dental Research). Grey literature was explored in a targeted manner, limited to professional society guidelines, position statements, and relevant conference abstracts identified through Google Scholar and organizational websites; exhaustive grey literature searches were not performed to avoid overrepresentation of non-peer-reviewed evidence. Inclusion prioritized randomized controlled trials, prospective cohorts, systematic reviews, and expert consensus documents published within the last decade. Case reports were excluded unless rare, but critical complications were observed. Two independent reviewers screened the titles and abstracts, and discrepancies were resolved by consensus.

Inclusion criteria: Randomized controlled trials, prospective or retrospective cohort studies, systematic reviews, meta-analyses, clinical guidelines, and expert consensus documents examining perioperative dental management of patients on NOAC/DOAC therapy. Exclusion criteria: Case reports (unless illustrating rare but clinically critical complications), in vitro or animal-only studies, pharmacokinetic studies unrelated to dental procedures, articles without perioperative relevance, and publications lacking sufficient methodological detail.

The initial search yielded 412 records after duplicates. Following title and abstract screening, 148 articles were selected for full-text assessment. Of these, 86 articles met the inclusion criteria, comprising randomized controlled trials, prospective cohorts, systematic reviews, and expert consensus documents, while 62 were excluded for reasons such as insufficient relevance, case-report-only evidence, or lack of NOAC-specific perioperative data. This narrative review was not conducted as a full Preferred Reporting Items for Systematic Reviews and Meta-Analyses (PRISMA)-compliant systematic review; however, core PRISMA principles, including structured database searching, predefined eligibility criteria, dual independent screening, and transparent reporting of article selection, were followed to enhance rigor and reproducibility.

Pharmacological profile of NOACs

The pharmacological profile of NOACs underpins their perioperative management in the dental setting. Dabigatran, a direct thrombin inhibitor, rapidly reaches peak plasma levels approximately 1-2 hours after ingestion, making the timing of the most recent dose and relies heavily on renal excretion, making its duration of action particularly sensitive to kidney function. In contrast, factor Xa inhibitors such as rivaroxaban, apixaban, and edoxaban exhibit varying degrees of hepatic metabolism and biliary clearance, with apixaban demonstrating the least renal dependence [6]. This heterogeneity influences the timing of dose omission prior to the dental procedures. For patients with normal renal function, holding a single morning dose of a twice-daily agent such as apixaban typically allows sufficient drug clearance the following morning, whereas once-daily agents such as rivaroxaban may require a longer interval to reach trough levels conducive to hemostasis [8]. Clinicians must account for these kinetic differences when scheduling procedures, ideally aligning interventions with periods of minimal residual anticoagulation, such as the early morning following an evening dose skip (Table 1). Renal impairment, prevalent among older adults seeking dental care, extends elimination half-lives and necessitates longer preoperative pauses or dose adjustments, highlighting the importance of preoperative creatinine clearance assessment [9].

**Table 1:** Expanded pharmacokinetic and clinical summary of NOACs Tmax: time to reach maximum plasma concentration; P-gp: P-glycoprotein transporter protein; CYP3A4/3A5/2J2: cytochrome P450 enzyme family involved in drug metabolism; CES1: Carboxylesterase 1 enzyme (involved in edoxaban metabolism); CrCl: creatinine clearance; AUC: area under the concentration-time curve; Cmax: maximum plasma concentration; BID/OD: twice/once daily dosing; NOACs: novel oral anticoagulants

Agent	Absorption/bioavailability	Tmax (time to peak)	Hepatic metabolism (%)	Contraindications	Key drug–drug interaction notes (P-gp/CYP3A4, etc.)
Dabigatran	~6-7% (as prodrug dabigatran etexilate)	~1-2 h	Minimal CYP450; ~80% renal elimination	Active bleeding; mechanical prosthetic valves; severe renal impairment (CrCl < 30 mL/min)	P-gp substrate; avoid strong P-gp inhibitors (ketoconazole, quinidine) and inducers (rifampicin)
Rivaroxaban	~80-100% with food (10–20 mg doses)	~2.5-4 h	~32% via CYP3A4/3A5/2J2; ~14% non-CYP hydrolysis	Active bleeding; hepatic disease with coagulopathy; pregnancy/lactation	P-gp + CYP3A4 substrate; avoid strong dual inhibitors/inducers (ketoconazole, ritonavir, carbamazepine)
Apixaban	~50% (43-46%)	~3-4 h	CYP3A4/5 (moderate) + ~25% renal	Active bleeding; severe hepatic impairment (Child-Pugh C); caution in renal failure	P-gp + CYP3A4/5 substrate; avoid strong inhibitors (clarithromycin, itraconazole) or inducers (phenytoin, rifampicin)
Edoxaban	~62%	~1-2 h	Minimal CYP450; mainly hydrolysis (CES1, CYP3A4); ~50% renal	Active bleeding; hepatic disease with coagulopathy; pregnancy/lactation	P-gp substrate; ↑ exposure with inhibitors (cyclosporin, dronedarone); ↓ with inducers (phenytoin, carbamazepine)

Risk stratification

Risk stratification forms the foundation for safe perioperative decision-making. Thrombotic risk is primarily evaluated using the CHA₂DS₂-VASc score in patients with nonvalvular atrial fibrillation, where higher scores indicate greater vulnerability to stroke during brief anticoagulant interruptions [10]. Patients with recent venous thromboembolism or certain hypercoagulable states warrant heightened caution, as even short discontinuation periods can precipitate clot formation. On the bleeding side, dental procedures vary widely in terms of hemorrhagic potential. Simple restorative work or supragingival scaling poses a minimal threat, whereas surgical extractions, multiple implant placements, or extensive periodontal flap surgeries elevate risk through greater tissue trauma and vascular exposure [9]. Although the hypertension, abnormal renal and/or liver function, stroke, bleeding history, labile international normalized ratio, elderly (age >65), and drugs and/or alcohol (HAS-BLED) score was developed for spontaneous bleeding prediction, its components, such as hypertension, renal dysfunction, and prior hemorrhage, overlap with procedural risk factors and provide a useful, albeit imperfect, framework [11].

Local anatomical considerations, such as highly vascularized posterior regions or inflamed tissues, often amplify bleeding propensity beyond systemic predictors. Integrating patient-specific thrombotic profiles with procedure-related bleeding likelihood enables tailored strategies to avoid both excessive anticoagulation and unnecessary thromboprophylactic gaps. According to Curto et al. [12], interruption of edoxaban is generally not necessary in patients requiring minor oral surgery procedures. Kwak et al. [13] performed a retrospective study on 120 patients who were taking NOACs and concluded that most dental treatments can be performed safely in patients without an increased likelihood of bleeding. The evidence is generally observational, with limited randomized dental studies.

Evidence on continuation vs. interruption

Evidence increasingly supports the use of NOACs for low- to moderate-risk dental procedures. Observational data from large registries demonstrate that uninterrupted therapy rarely leads to severe haemorrhage [14]. In a prospective study involving patients on NOACs undergoing tooth extractions, clinically significant bleeding occurred in <10% of cases (6.9%), all managed conservatively without transfusion or hospitalization [15]. Thromboembolic complications were absent, underscoring the safety of anticoagulation maintenance when local hemostatic measures were employed.

Similar findings were reported in a previous study of 49 patients taking rivaroxaban, apixaban, edoxaban, and dabigatran. Bleeding episodes were more frequent in patients treated with rivaroxaban and apixaban [16]. Randomized controlled trials remain limited, but available comparisons between continued and briefly interrupted regimens reveal no meaningful differences in major bleeding, although minor seepage is modestly increased with ongoing therapy [1,2,6,9].

López-Galindo and Grau-Benítez [17] conducted a systematic review and included seven studies comprising 931 patients. It was concluded that the predominant postoperative complication observed in patients administered NOACs and those receiving vitamin K antagonists following uncomplicated dental extractions was minor bleeding, which may occur either immediately or subsequently. Evidence suggests that NOACs are a relatively safe pharmacological option and do not necessitate interruption or modification of the therapeutic regimen for individuals undergoing simple dental extractions.

When interruption is deemed necessary for higher-risk surgeries, a minimalist approach, such as omitting one or two doses timed to procedural trough levels, restores adequate hemostasis without bridging anticoagulation, which carries its own hemorrhagic burden [18]. Miclotte et al. [19] conducted a prospective study on 26 patients treated with dabigatran, rivaroxaban, or apixaban and compared them with 26 controls. It was concluded that if the morning dose of NOAC is skipped, excessive bleeding during and after the procedure can be avoided.

Local hemostatic measures

Local hemostatic interventions are pivotal in mitigating bleeding when NOACs are continued. Tranexamic acid, when applied as a topical mouthwash, inhibits fibrinolysis at the surgical site with minimal systemic absorption [20]. A systematic review showed that patients administered local tranexamic acid therapy for postoperative bleeding reduction in dental procedures had a 7% lower risk of oral bleeding. Absorbable hemostatic agents, including gelatin sponges and oxidized cellulose, provide mechanical barriers within extraction sockets and promote clot retention and stabilization [21]. Combining these materials with tranexamic acid-soaked gauze enhances efficacy, with near-elimination of rebleeding episodes in anticoagulated individuals [22]. Primary closure with resorbable sutures and prolonged local pressure further minimizes dead space and reinforces hemostasis. These adjuncts are inexpensive, widely available, and integrate seamlessly into routine dental practice, allowing clinicians to proceed with anticoagulation therapy for most interventions [23].

Minimally invasive techniques

Minimally invasive surgical techniques further tilt the bleeding-thrombosis balance toward safety. Flapless implant placement avoids periosteal reflection and extensive soft tissue dissection and preserves vascular integrity [24]. Piezoelectric instrumentation employs ultrasonic vibrations for precise bone cutting, spares surrounding soft tissues, and reduces intraoperative blood loss compared with conventional rotary methods [25]. Previous randomized controlled trial comparisons in third molar surgery highlighted significantly less postoperative swelling and oozing with this modality [26]. Laser-assisted procedures utilizing diode or erbium wavelengths achieve simultaneous cutting and coagulation through photothermal effects [27]. Although the evidence is preliminary, small studies have suggested reduced bleeding during soft-tissue surgeries [28]. Campos et al. [29] reported no bleeding during and after diode laser surgery in anticoagulated rats. These techniques require operator training, but offer substantial hemostatic advantages without necessitating systemic anticoagulant modification.

Reversal agents

Specific reversal agents, but transformative agents for life-threatening hemorrhage, have limited utility in dentistry. The necessity for reversal of the anticoagulant effects of NOACs may arise in patients experiencing significant hemorrhage and in those requiring immediate surgical intervention or other urgent procedures. Reversal can be achieved through the administration of specific reversal agents, including idarucizumab for dabigatran and andexanet alfa for apixaban, edoxaban, and rivaroxaban, or through the use of nonspecific agents, such as prothrombin complex concentrates, activated prothrombin complex concentrate, and recombinant activated factor VII [30].

Idarucizumab rapidly neutralizes dabigatran by binding to its active site, restoring thrombin generation within minutes [31]. Full-cohort analyses from idarucizumab reversal trials involved patients presenting with major bleeding events, including intracranial hemorrhage, gastrointestinal bleeding, trauma-related bleeding, and postoperative surgical bleeding, and demonstrated rapid hemostatic efficacy in these severe settings; however, dental-specific use remains anecdotal and is reserved only for uncontrolled postoperative oral bleeding that does not respond to local hemostatic measures [32]. Ciraparantag reverses the anticoagulant activity of apixaban and rivaroxaban [33], with clinical studies demonstrating prompt normalization of coagulation parameters [34]. However, its high cost, short duration of action, and rebound anticoagulation potential restrict its use in emergency situations. Prothrombin complex concentrates offer an alternative to factor Xa inhibitors when andexanet is unavailable, but evidence in dental contexts is sparse [35]. Routine reversal before elective procedures is unwarranted and potentially pro-thrombotic.

Multidisciplinary collaboration

Multidisciplinary collaboration enhances perioperative outcomes. Preoperative consultation with the prescribing physician clarified thrombotic risk and renal status, informing interruption decisions. Hematological input is invaluable for high-risk patients or those with recent thromboembolic events. Postoperative follow-up protocols, including clear instructions on hemostatic rinses and early recall for suture removal, can prevent delayed complications. Patient education on the signs of excessive bleeding or thrombosis empowers timely intervention. Institutional protocols standardize NOAC management and streamline care across providers [36].

Clinical implications

**Figure 1:**
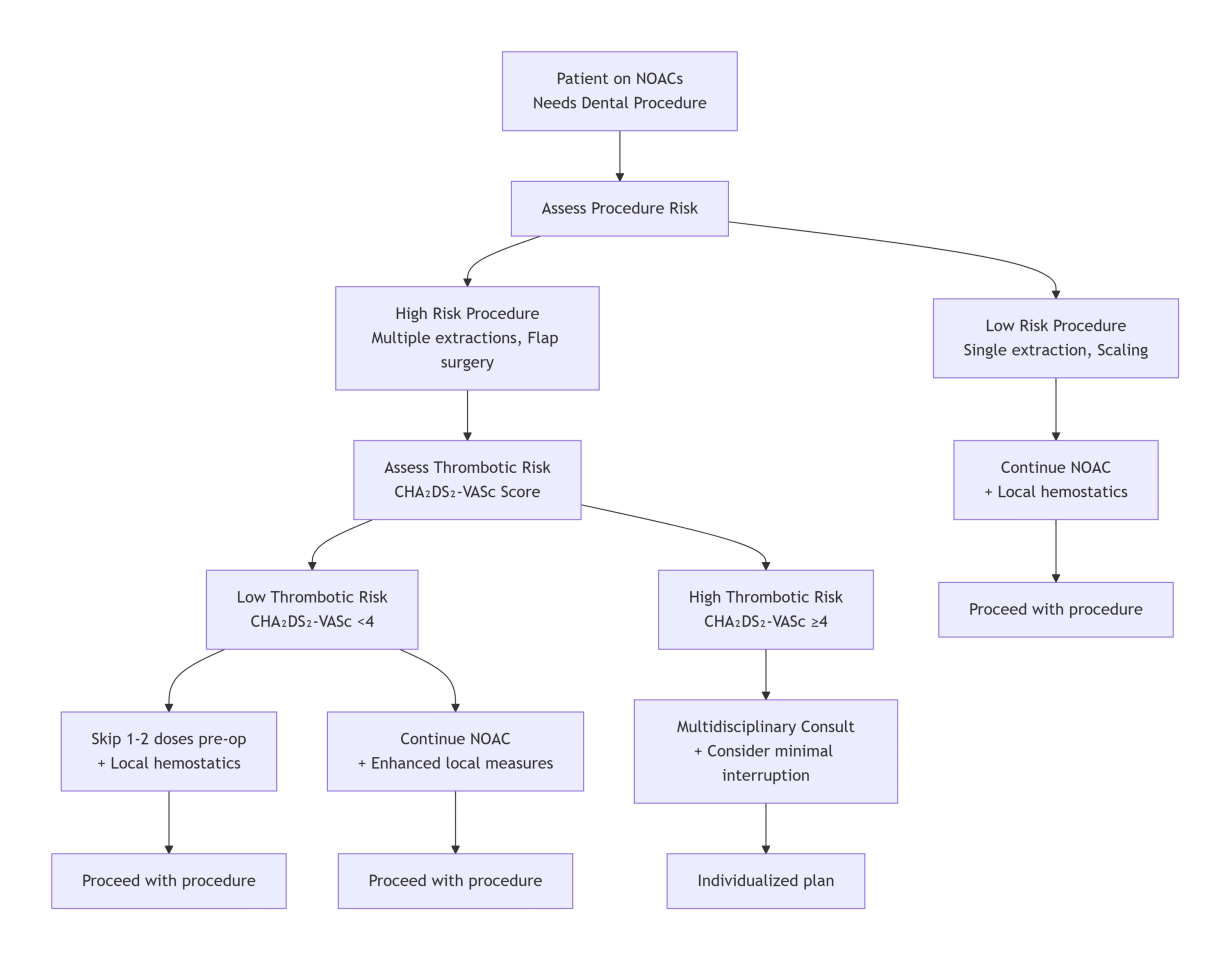
Clinical decision algorithm for dental management in patients taking novel anticoagulants (NOACs) The image was created using Microsoft® PowerPoint® 2021 (Microsoft Corporation, Redmond, WA, USA). Image credit: Dr Rama Shankar Choudhary

The clinical implications of this evidence are important in daily dental practice. For low-bleeding-risk procedures, such as single extractions or routine restorations, NOACs should be continued with local hemostatic adjuncts, such as tranexamic acid mouthwash and absorbable agents. Moderate-risk surgeries, including multiple extractions or single implants, may proceed uninterrupted in patients with low thrombotic risk, whereas those with elevated CHA₂DS₂-VASc scores benefit from a brief dose omission timed to trough levels, supplemented by robust local measures. High-risk extensive surgeries necessitate multidisciplinary planning, potentially including 24-to 48-hour preoperative pauses and postoperative tranexamic acid prophylaxis. Minimally invasive techniques should be employed whenever feasible to minimize tissue trauma. Reversal agents are reserved for rare uncontrolled bleeding episodes (Figure 1).

Recommendations

Recommendations include routine preoperative renal function assessment and thrombotic risk scoring. Procedures should be scheduled in the morning after omitting the prior evening dose of once-daily agents. Local hemostatics such as tranexamic acid, gelatin sponges, and primary closure form the first-line defense against bleeding. Flapless and piezoelectric approaches are preferred for implants and surgical extraction. Clear postoperative instructions and 24-to 48-hour follow-up reduced rebleeding. Reversal agents are not indicated prophylactically but should be accessible in facilities managing high-risk patients. A summary of NOACs has been given in Table 2.

**Table 2:** Summary of commonly used novel oral anticoagulants (NOACs) BID: twice daily dosing; OD: once daily dosing; CrCl: creatinine clearance; PCC: prothrombin complex concentrate; VTE: venous thromboembolism; AF: atrial fibrillation

Agent	Class/target	Typical adult dosing (common)	Half-life (approx.)	Renal clearance/dependence	Oral bioavailability	Dosing frequency	Reversal agent	Key trial/reference	Quick dental/clinical note
Dabigatran	Direct thrombin (IIa) inhibitor	150 mg BID (110 mg BID or 75 mg BID in some cases)	12-17 h	High (~80%) renal	~6-7% (prodrug)	BID	Idarucizumab	RE-LY [7]	Renal function critical; longer pre-operative pause if CrCl reduced
Rivaroxaban	Factor Xa inhibitor	20 mg OD (15 mg OD in moderate renal impairment/15 mg BID initial for VTE)	5-13 h	Moderate (~33%)	~80-100% with food	OD	Andexanet alfa or PCC	ROCKET-AF [37]	Once-daily dosing; schedule at trough; local hemostatics are effective
Apixaban	Factor Xa inhibitor	5 mg BID (2.5 mg BID if dose-reduction criteria)	~12 h	Low (~25%)	~50%	BID	Andexanet alfa or PCC	ARISTOTLE [38]	Least renal dependence; dental work is often possible without interruption
Edoxaban	Factor Xa inhibitor	60 mg OD (30 mg OD if CrCl 15-50 mL/min or low weight)	10-14 h	Moderate (~50%)	~62%	OD	Andexanet alfa or PCC	ENGAGE AF-TIMI 48 [39]	Once-daily agent; adjust dose by renal function and skip prior dose for high-risk procedures

Limitations

**Figure 2:**
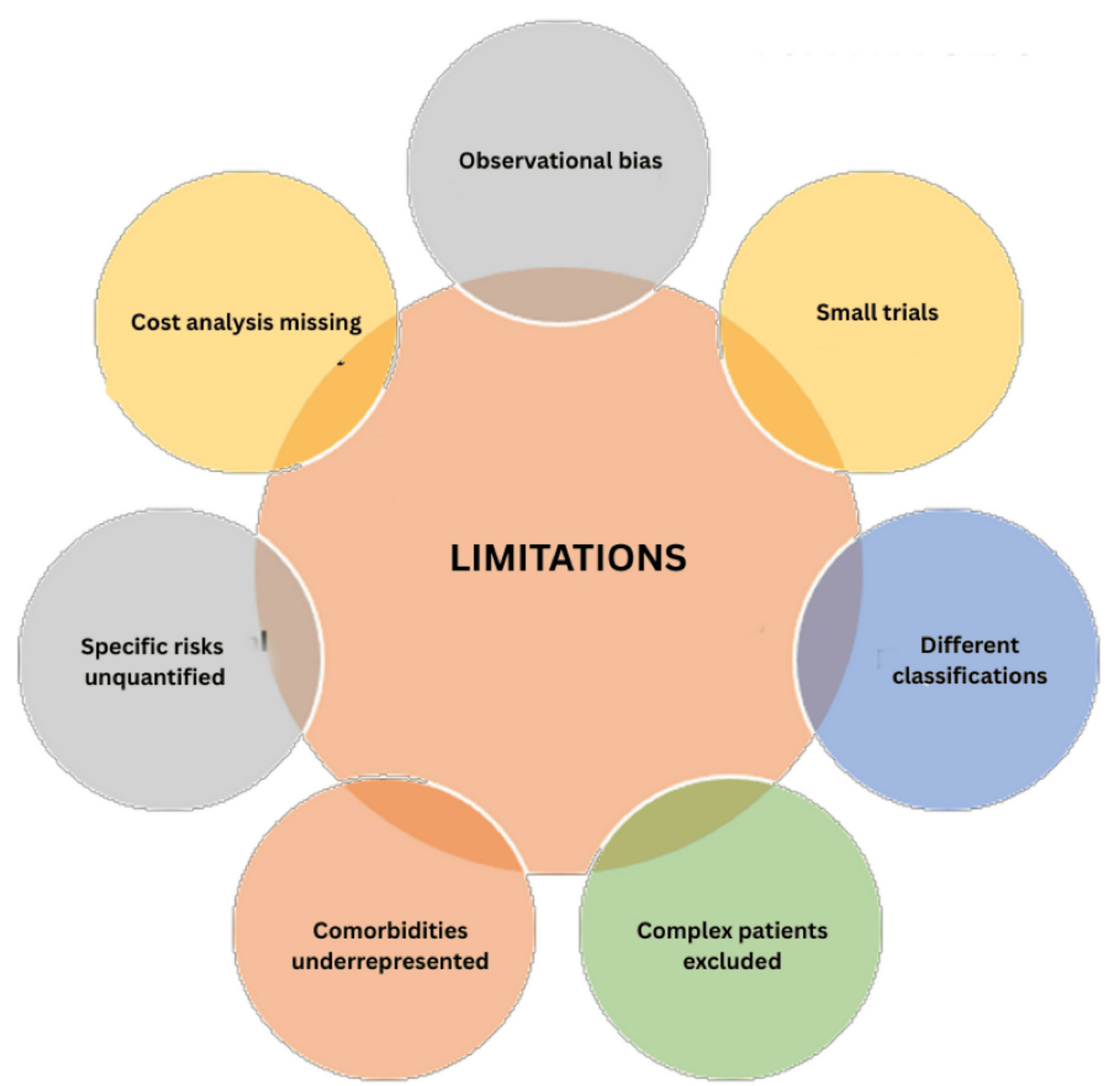
Limitations of novel oral anticoagulants (NOACs) The image was created and edited using Adobe Photoshop version 7.0 (Adobe Systems, San Jose, CA, USA). Image credit: Dr Rama Shankar Choudhary

The limitations of the evidence base tempered these recommendations. Most of the studies were observational, with a few randomized trials powered for dental endpoints. Heterogeneity in procedural definitions, bleeding classifications, and NOAC regimens complicates comparisons. Long-term thrombotic outcomes after brief interruptions remain underreported. Renal impairment, polypharmacy, and antiplatelet co-administration, which are common in real-world patients, are underrepresented in clinical trials. Procedure-specific bleeding risks lack granular quantification, and the cost-effectiveness of adjunctive measures has not been evaluated. Future research should prioritize prospective registries with standardized outcomes and randomized comparisons of continuation versus tailored interruption strategies (Figure 2).

## Conclusions

In summary, the management of patients taking NOACs undergoing dental procedures requires a nuanced, evidence-based approach to harmonize bleeding and thrombotic risks. Continuation of NOACs with robust local hemostatic measures such as tranexamic acid rinses, absorbable agents, and primary closure safely facilitates low- to moderate-risk interventions, obviating unnecessary interruptions that increase stroke vulnerability. For higher-risk surgeries, brief, timed dose omissions aligned with pharmacokinetic troughs coupled with minimally invasive techniques such as flapless implants and piezoelectric surgery optimize hemostasis without compromising thromboprophylaxis. Reversal agents remain the last resort for uncontrolled hemorrhage. Multidisciplinary risk stratification using CHA₂DS₂-VASc and procedural bleeding profiles, along with renal function assessment, underpins patient-centered protocols. Despite evidentiary gaps in randomized data and procedure-specific outcomes, current strategies empower clinicians to deliver safe oral healthcare. Future prospective studies should refine interruption thresholds and validate adjunctive technologies to ensure sustained equilibrium in this burgeoning anticoagulation population.
